# Deep learning networks-based tomato disease and pest detection: a first review of research studies using real field datasets

**DOI:** 10.3389/fpls.2024.1493322

**Published:** 2024-10-25

**Authors:** Mohieddine Jelali

**Affiliations:** Cologne Lab for Artificial Intelligence and Smart Automation (CAISA), Institute of Product Development and Engineering Design (IPK), TH Köln – University of Applied Sciences, Cologne, Germany

**Keywords:** convolutional neural networks, deep learning, plant disease detection, object detection, tomato datasets

## Abstract

Recent advances in deep neural networks in terms of convolutional neural networks (CNNs) have enabled researchers to significantly improve the accuracy and speed of object recognition systems and their application to plant disease and pest detection and diagnosis. This paper presents the first comprehensive review and analysis of deep learning approaches for disease and pest detection in tomato plants, using self-collected field-based and benchmarking datasets extracted from real agricultural scenarios. The review shows that only a few studies available in the literature used data from real agricultural fields such as the PlantDoc dataset. The paper also reveals overoptimistic results of the huge number of studies in the literature that used the PlantVillage dataset collected under (controlled) laboratory conditions. This finding is consistent with the characteristics of the dataset, which consists of leaf images with a uniform background. The uniformity of the background images facilitates object detection and classification, resulting in higher performance-metric values for the models. However, such models are not very useful in agricultural practice, and it remains desirable to establish large datasets of plant diseases under real conditions. With some of the self-generated datasets from real agricultural fields reviewed in this paper, high performance values above 90% can be achieved by applying different (improved) CNN architectures such as Faster R-CNN and YOLO.

## 1 Introduction

The agricultural sector is of vital importance in maintaining human life and providing essential food resources. Plant health and accurate and timely diagnosis of plant diseases are of great importance for human health and the economy of the farmers in the world. The issue of plant diseases and pests represents a significant challenge for the agricultural sector. However, plant diseases and pests represent a significant threat to crop yield and quality, leading to substantial economic losses and food insecurity. Among various crops, tomatoes are particularly vulnerable to a wide range of diseases caused by fungi, bacteria, viruses, and environmental stressors. The effective detection of plant diseases can reduce output losses and ensure the long-term sustainability of agricultural production. This represents a significant challenge for the implementation of agricultural informatization and intelligent industry, as well as the realization of high-quality, efficient, and safe agricultural production (Gong and Zhang, 2023).

Tomatoes are one of the most commonly consumed fruits on a daily basis. As tomatoes are utilized in a variety of condiments, including ketchup, sauce, and puree, their global utilization rate is high. They constitute approximately fifteen percent (15%) of all vegetables and fruits, with an annual per capita consumption of twenty kilograms. In Europe, an individual consumes approximately 31 kg of tomatoes per year. The high demand for tomatoes necessitates the development of early detection technologies for viruses, bacteria, and viral contaminations (Shoaib et al., 2022).

The advent of new technologies has opened up promising avenues for tackling this challenge. Deep learning (DL), a subset of artificial intelligence (AI), has emerged as a powerful tool for image analysis and pattern recognition. Convolutional neural networks (CNNs), a popular architecture in DL, have demonstrated remarkable success in a range of image classification tasks, including object detection, fruit counting, automated harvesting, and, notably, plant disease detection and diagnosis. Among ML and DL techniques, CNNs are frequently the preferred choice for image detection and classification due to their intrinsic capacity to autonomously acquire pertinent image features and comprehend spatial hierarchies. CNN algorithms are well-suited for the classification of plant diseases, offering flexibility and a feature extractor property that enables the automated extraction of features Demilie (2024).

The field of object detection technology represents a significant area of current research interest. It has been developed to address the shortcomings of traditional methods of disease diagnosis. The technology is capable of classifying and defining the category of objects by locating and predicting the position of objects in images or videos. Object detection algorithms are primarily classified into two categories: two-stage algorithms and one-stage algorithms. Two-stage algorithms, exemplified by ResNet, LeNet-5, AlexNet, GoogLeNet, and Faster R-CNN, comprise a two-step process, whereas one-stage algorithms, exemplified by SSD, VGG, and YOLO, comprise a single step. Two-step algorithms exhibit relatively high accuracy but are constrained by computational demands and real-time performance (Orchi et al., 2023).

A considerable number of reviews has been published on the current status of DL applications in object detection and crop disease image recognition. An earlier review was presented in (Ngugi et al., 2021). This paper presents a comparative analysis of the performance of ten deep learning models on the task of leaf disease recognition using the well-known PlantVillage dataset (Hughes and Salathe, 2015). The review by Darwin et al. (2021) highlighted the merits and demerits of different machine vision and deep learning techniques along with their various performance metrics. The authors stated that DL models outperform conventional image processing techniques with an average accuracy of 92.51% in diverse agricultural applications. The work of Li et al. (2021) reviewed studies conducted in the field of plant leaf disease detection to identify plant leaf diseases using image processing, hyperspectral imaging, and DL techniques. Publications from 2019–2020 were considered by the authors. They discussed the importance of collecting large datasets with high variability, data augmentation, transfer learning, and visualization of CNN activation maps in improving classification accuracy, as well as the importance of detecting plant leaf diseases with small samples and the importance of hyperspectral imaging for early detection of plant diseases. Liu and Wang (2021) delineated research on plant disease and pest detection using DL, focusing on three aspects: classification networks, detection networks, and segmentation networks. Additionally, the paper summarized the advantages and disadvantages of each method. The review in (Dhaka et al., 2021) undertook a comparative analysis of the pre-processing techniques, CNN models, frameworks, and optimization techniques applied to detect and classify plant diseases using leaf images as a dataset. This paper also presented a survey of the datasets and performance metrics used to evaluate the efficacy of models. A total of 100 publications were reviewed by Tugrul et al. (2022) based on detection methods and model performance evaluation, comparison of popular CNN frameworks, detailed description of CNN applications in agricultural fields, dataset preparation, the problem and solution related to plant leaf disease detection, and publicly released datasets in the relevant field.

The paper by Salman et al. (2023) provides a bird’s eye view of plant disease datasets, deep learning techniques, their accuracies, and challenges. A comprehensive review of the latest developments in object detection algorithms and the underlying concepts behind these methods is provided by Amjoud and Amrouch (2023). The paper by Zou et al. (2023) provides an exhaustive review of the advancements in object detection over the past two decades (1990–2022). It provides a comprehensive analysis of seminal detectors, pivotal technologies, accelerated methodologies, datasets, and performance metrics. Another survey of 70 studies on the applications of deep learning (DL) and the trends associated with their use for disease diagnosis and management in agriculture was presented in reference to the paper by (Ahmad et al., 2023b). A review of recent research on deep learning models-based plant disease detection was provided by Shoaib et al. (2023). The authors focused on publications between 2015 and 2022. In (Attri et al., 2023), 129 papers (2017–2022) that employ DL applications in agricultural contexts were examined and classified into five categories: crop yield prediction, plant stress detection, weed and pest detection, disease detection, and smart farming. The study by Doutoum and Tugrul (2023) presents a review of leaf disease research in the literature. The total number of papers retrieved from five electronic databases was 256. Ramanjot et al. (2023) analyzed existing techniques in terms of data sources, pre-processing techniques, feature extraction techniques, data augmentation techniques, models utilized for detecting and classifying diseases affecting the plant, image enhancement techniques, overfitting reduction techniques, and accuracy. Peer-reviewed publications from various databases published between 2010 and 2022 were selected as research papers for this study. The paper of Zhou et al. (2023) reviewed the application of object detection methods to the recognition of common plant diseases, such as tomato, citrus, corn, and pine. It presents various object detection models, from basic to advanced and sophisticated networks, and compares the innovative aspects and drawbacks of commonly used neural network models.

A recent review of crop disease detection with DL was offered by Ngugi et al. (2024). The review examines the performance analysis of the latest machine learning (ML) and DL techniques outlined in the literature. Furthermore, the study reviewed recent research initiatives, providing an overview of publicly accessible datasets pertaining to plant diseases. The review by Odounfa et al. (2024) has provided a detailed analysis of the current state of deep learning methods employed for the detection and severity estimation of stresses and pests affecting market garden crops, such as tomatoes, cucumbers, peppers, and leafy greens. A survey of 135 articles published between 2017 and 2023 on ML and DL image-based plant disease classification for industrial farming systems was conducted by Sajitha et al. (2024). Various aspects of these systems, including the sources of plant datasets, algorithm types and techniques, are examined.

The most comprehensive, recent, and impressive review is that of Demilie (2024), who considered 161 publications. The author summarized the research on disease detection and classification in plant leaves and crops using deep learning and machine learning, including the type of plant, techniques/models/algorithms used, and accuracy. The review revealed that the majority of the included studies focused on the detection and classification of diseases in tomato (39%) and rice crops (16%), respectively. This suggests that the tomato plant is the most extensively researched plant leaf and/or crop species, highlighting its susceptibility to stress and pest-related challenges. This prominence can be attributed to the economic significance of the species and its widespread cultivation (Odounfa et al., 2024). Saranya et al. (2021) surveyed 38 research works that applied DL techniques to various research problems in tomato plant. The paper by Domingues et al. (2022) presented a literature review on ML techniques used in the agricultural sector, with a particular focus on the tasks of classification, detection, and prediction of diseases and pests, with an emphasis on tomato crops.

The main contribution of this paper is providing the first review of methods and application studies for the detection of diseases and pests in tomato plants using DL, based on field databases. The objective is to develop a system for classifying plant diseases based on the analysis of leaf images collected under real-field conditions. The objective of the studies reviewed in this paper is not to classify the leaves in the images according to their species or plant type, as this information is already known *a priori* in real agricultural situations. Instead, the aim is to identify and classify the disease or pest present in the images.

## 2 Scope and methodology of the review

In the last decade, CNNs have been widely adopted in the field of image-based disease and pest detection in plants. The present review focuses on the use of DL methods for the detection of *multiple* diseases and pests affecting tomato plants (leafs). Although a considerable portion of studies have validated DL-based image recognition models on datasets comprising multiple crop classification and disease identification, there is a growing interest in developing deep image-based disease diagnostic models for a specific crop, assuming that farmers are already aware of the type of crop they are cultivating (Khatoon et al., 2021). This *disease classification* task aims to predict the diseases of plants from leaf images. Note that “healthy” is also treated as a category of plant diseases. The task will not only identify whether a plant has a disease or not but also need to accurately categorise the disease types from different plants. A model should be able to focus on the diseases and not be confused by the common patterns from leaves of the same type (Yao et al., 2024).

**Figure 1:**
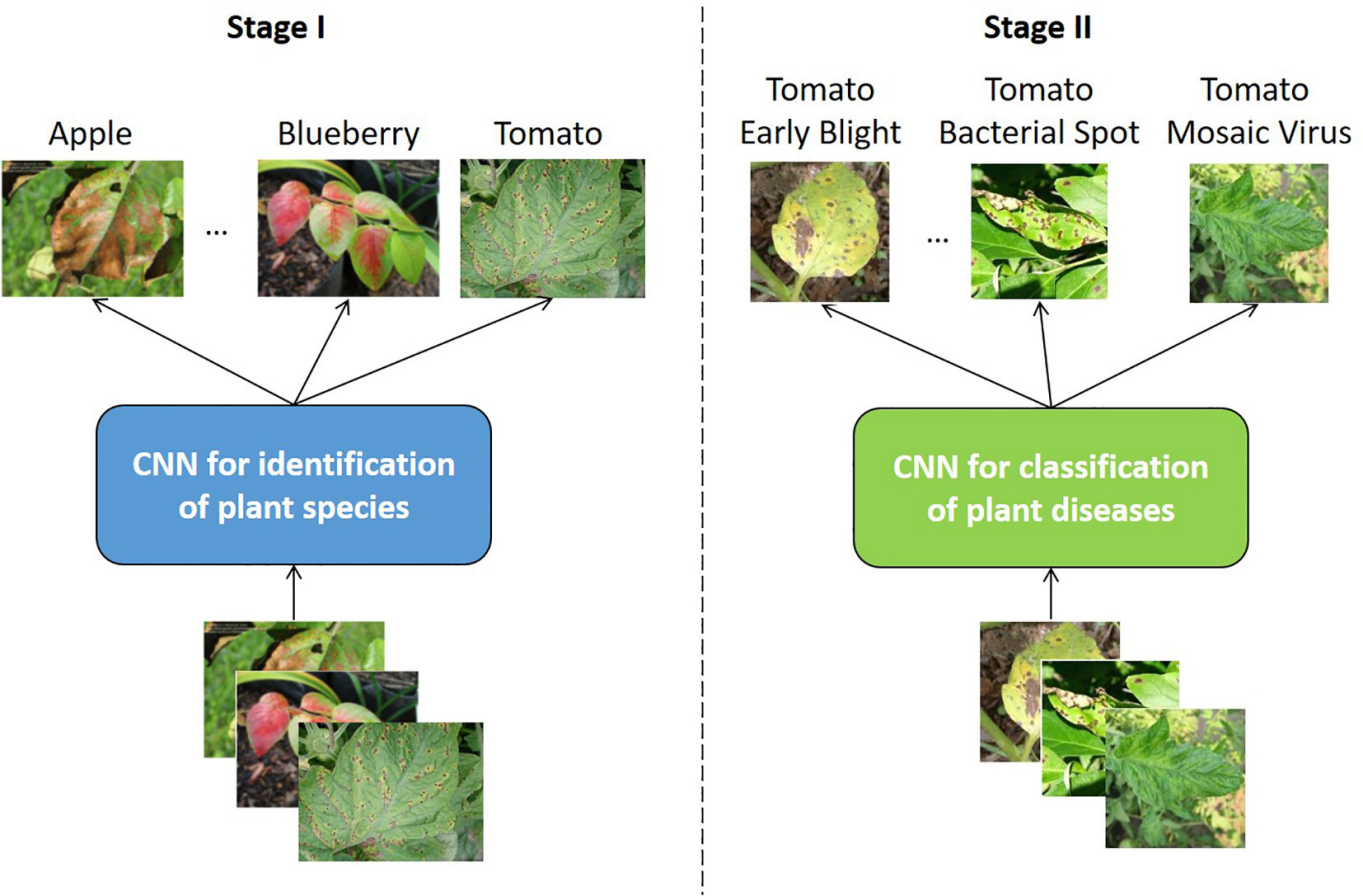
Separate plant species identification (Stage I) and disease classification processes (Stage II) using independent CNNs.

To guarantee the reliability of the analysis, it is crucial that only studies incorporating data exclusively collected for the tomato species are included. It is therefore imperative that researchers refrain from attempting to identify the plant type itself from a multi-plant dataset. The rationale for this is that the present work is primarily concerned with object recognition models for monitoring the cultivation of crops, for example, those cultivated hydroponically, as demonstrated in the work by Schneider et al. (2024). When considering the practical application of such a model, it becomes evident that the specific crop currently being cultivated is typically known. In a hydroponic system, for instance, a single plant is typically cultivated in a given cycle. It is therefore unnecessary for a model designed for monitoring these plants to be capable of differentiating between different species, as the plant species is always known *a priori*. The complexity of a model increases in direct proportion to the number of classes in a dataset that require differentiation. Furthermore, an increased number of classes can also have a negative effect on the accuracy of the model, consequently affecting its metrics. From a practical standpoint, therefore, the simultaneous prediction of species and diseases is unnecessary. It can be regarded as an academic exercise at most. Even in the rare cases where the plant species needs to be identified, it is best to take a two-stage approach, i.e. to use two independent CNNs, one for predicting plant species and the other for predicting disease types within the same plant species; see **Figure 1**. This approach separates the two tasks and thus overcomes the problem of the growth of computational complexity associated with the simultaneous identification of species and diseases.

Our search, conducted between 2016 and 2024, identified 35 relevant articles. Of these, 27 were original journal publications, 6 were conference proceedings, 1 was a preprint (Research Square), and 1 was an arXiv article. We chose 2016 to start the search because it was the year following the publication of the prominent PlantVillage dataset. In addition, 2016 was the year that Mohanty et al. (2016) published one of the most cited papers on the topic. In the following analysis, we focus on papers that address the detection of tomato leaf diseases and pests using *(at least partially) real field datasets*. Papers will not be considered for review in Section 4 if they use only PlantVillage or analogous datasets for benchmarking purposes.

## 3 Review of agriculture datasets

The main problem faced by most researchers in this field is the lack of available datasets. This would greatly affect and restrict the research of machine learning for plant identification and disease classification from leaf images. Fortunately, in recent years, several attempts have been made successfully and researchers have devoted themselves to the collection of plant disease data, filling the data availability gap in this area (Chouhan et al., 2020; Yao et al., 2024). While some of the researchers have worked with the database provided by a laboratory or research organization, others have evaluated their work using a self-generated database. PlantVillage, PlantDoc, IP102, Flavia, and MalayaKew Leaf are among the datasets that are publicly available.

The pathology for tomato diseases is complex, and the symptom may manifest on fruit or leaves. Some common diseases include bacterial spot, which can affect fruit, leaf, and stem, and bacterial canker, which appears on the crown and above. Other diseases include early blight, late blight, leaf mold, and septoria leaf spot, which are caused by Alternaria fungus, Phytophthora infestans, Passalora fulva, and Septoria lycopersici, respectively (Zhou et al., 2023; Cornell University, 2024). For descriptions of plant diseases and management practices affecting specific crops, including tomatoes, the reader is referred to (Cornell University, 2024).

The following is a concise overview of five publicly available datasets used in many studies (Domingues et al., 2022; Yao et al., 2024):

- *PlantVillage* (Hughes and Salathe, 2015): The PlantVillage dataset has been one of the most frequently utilized public datasets for research on the identification and classification of leaf diseases for over a decade. The dataset is available in multiple versions, including an original version and a data augmentation version. The original dataset was first published in 2015 and comprises 54,305 images of diseased and healthy leaves from 14 plant species, including apple, blueberry, cherry, corn, grape, orange, peach, bell pepper, potato, raspberry, soybean, squash, strawberry, and tomato. Each species is associated with one to ten distinct disease categories, resulting in a total of 22 unique disease categories. It should be noted that some species exhibit a tendency to share several diseases. The dataset comprises a total of 38 unique combinations of species and diseases, in addition to an additional category of images lacking leaves (1,143 background images). The images utilized in Plant Village were captured in a laboratory setting, rather than in the actual conditions of cultivated fields, which could potentially influence the trained model’s effectiveness in real-world scenarios.
- *PlantDoc* and *Cropped PlantDoc* (Singh et al., 2020): The PlantDoc dataset is a non-laboratory-based collection of images and information pertaining to leaf disease detection. Most of the images included in the dataset depict diseases contracted in field conditions. However, the majority of these images were sourced from online sources, resulting in a highly variable dataset. The images feature a multitude of backgrounds, many of which are complex and diverse. The dataset contains images of similar categories of plant species and disease types as those found in PlantVillage, with 2,598 leaf images, 13 plant species, and 17 unique diseases. There are 38 classes for a combination of species and diseases. PlantDoc is thus considerably smaller than PlantVillage. In addition to the PlantDoc dataset, Singh et al. (2020) have also introduced a second dataset, namely the “Cropped PlantDoc Dataset”, by cropping the images with bounding box information. This dataset includes low-resolution cropped images of diseased and healthy leaves from the original PlantDoc dataset.
- *Taiwan Tomato* (Huang and Chang, 2020): This dataset contains 622 photographs of Taiwan tomato leaves categorized into six groups (five disease categories and one healthy category). It includes a single leaf, many leaves, simple background, and complicated background.
- *Tomato-Village* (Gehlot et al., 2023): This new database was created when Gehlot et al. attempted to predict tomato diseases in the field in the Jodhpur and Jaipur districts of Rajasthan, India, and found that the majority of diseases were leaf miner, spotted wilt virus, and nutrient deficiency diseases, but no public datasets were available that included these categories. Three variants of the dataset were then established for a) multiclass tomato disease classification, b) multilabel tomato disease classification, and c) object detection-based tomato disease detection. Using numerous plant pathology references, Internet resources, and local agricultural experts, labels were identified for 3,231 images taken from three different locations in Rajasthan. These images may contain one disease (belonging to one label) or many diseases (having multiple labels). Of the 3,231 images, 2,103 contain one disease, 1,106 contain two diseases, and 22 contain three diseases. The multiclass variant of the dataset contains 2,103 images with a single disease per image, as required for multiclass classification, while the multilabel variant contains all 3,231 images with either a single disease or multiple diseases per image. For the multilabel variant dataset, a CSV file was created with columns for image name and each disease category, such as early blight, healthy, late blight, leaf miner, magnesium deficiency, nitrogen deficiency, potassium deficiency, and spotted wilt virus (Gehlot et al., 2023).
- *FieldPlant* (Moupojou et al., 2023): FieldPlant is a relatively new plant disease dataset of 5,170 annotated field leaf images collected from plantations in Cameroon, the world’s tenth largest tomato producer. The dataset focuses on different diseases in three tropical crops: corn, cassava, and tomato. The manual annotation of individual leaves on each image was conducted under the supervision of plant pathologists to ensure the quality of the process.

**Figure 2:**
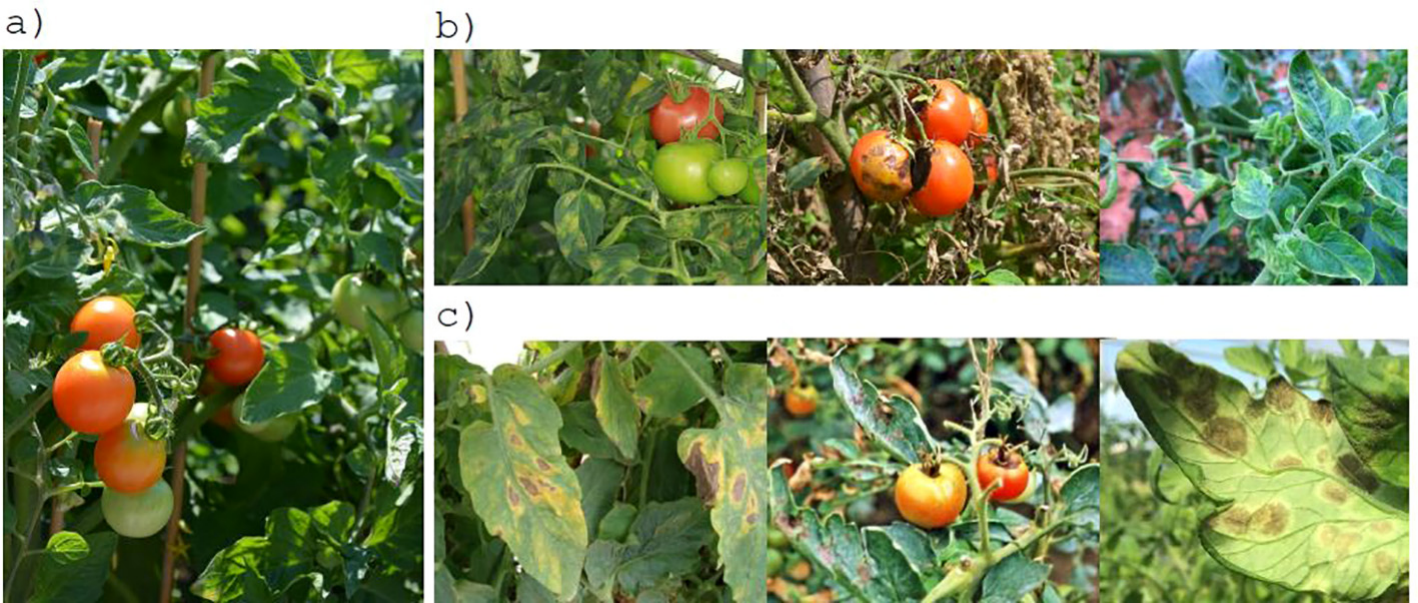
Challenges associated with detection and classification of tomato diseases and pests: **(A)** complex plant environments^1^, **(B)** multiple disease types^2^, **(C)** similarity among classes^3^.

**Table 1** shows the disease categories included in some selected tomato disease databases. A comprehensive survey of plant disease databases is presented in (Mondal et al., 2023). The majority of published studies have utilized image data obtained from the PlantVillage dataset. In a controlled laboratory setting, images are typically composed of a single leaf superimposed on a neutral artificial background. In contrast, images acquired in the field exhibit considerably greater complexity than those obtained in a laboratory setting. This is due to the presence of multiple leaves in a single image, the inclusion of additional plant parts, variations in shading and lighting, diverse ground textures, and different backgrounds; see **Figure 2**. Accordingly, the PlantVillage database is not an optimal choice for training deep learning models due to the prevalence of similar backgrounds and lighting conditions. Images obtained in a controlled laboratory setting and those acquired in the field can yield markedly disparate outcomes and processes (Domingues et al., 2022). The classification of diseases and pests is significantly more challenging when images are acquired in the field than in a controlled setting. PlantDocillustrates that cropping leaves enhances the precision of CNN architectures when processing in-field images (Singh et al., 2020). Consequently, it is unsurprising that numerous studies utilizing PlantVillage yield results with an accuracy exceeding 90%.

**Table 1:** Diseases/categories available in different tomato disease databases.

Tomato-related dataset	Tomato disease category	No. of images	
D1: PlantVillage	Bacterial Spot	2,127	
	Early Blight	1,000	
	Healthy Leaf	1,591	
	Late Blight	1,909	
	Leaf Mold	952	
	Septoria Leaf Spot	1,771	
	Spider Mites Two-spotted Spider Mite	1,676	
	Target Spot	1,404	
	Mosaic Virus	373	
	Yellow Leaf Curl Virus	5,357	
	**Total**	**18,160**	
		D2	D3
D2: PlantDoc	Bacterial Spot	110	273
D3: Cropped PlantDoc	Early Blight	88	207
	Healthy Leaf	63	391
	Late Blight	111	203
	Leaf Mold	91	291
	Septoria Leaf Spot	151	418
	Spider Mites Two-spotted Spider Mite	3	3
	Mosaic Virus	54	254
	Yellow Leaf Curl Virus	76	713
	**Total**	**747**	**2,753**
D4: Taiwan Tomato	Bacterial Spot	880	
	Black Mold	536	
	Gray Spot	672	
	Healthy	848	
	Late Blight	784	
	Powdery Mildew	1,256	
	**Total**	**4,976**	
D5: FieldPlant	Bacterial Wilt	2	
	Blight Leaf	410	
	Brown Spots	1,967	
	Healthy Leaf	279	
	Leaf Mosaic Virus	19	
	Leaf Yellow Virus	115	
	**Total**	**2,792**	
D6: Tomato-Village	Early Blight	62	
	Healthy Leaf	216	
	Late Blight	113	
	Leaf Miner	1,024	
	Magnesium Deficiency	117	
	Nitrogen Deficiency	45	
	Potassium Deficiency	9	
	Spotted Wilt	517	
	**Total**	**2,103**	
	2 diseases	1,106	
	3 diseases	22	
	**Total**	**3,231**	

**Figure 3:**
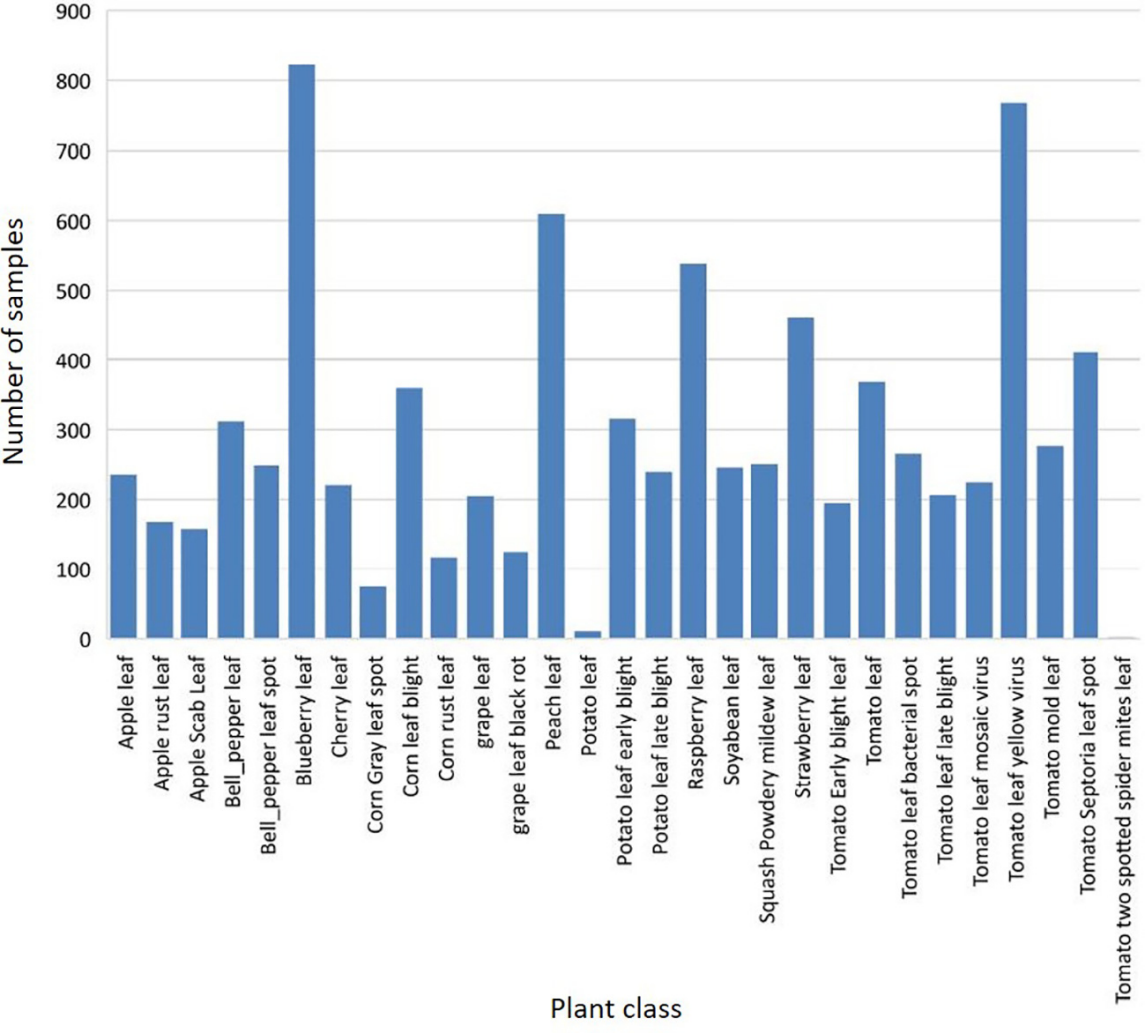
Sample (instance/bounding box) frequencies in PlantDoc dataset by plant classes.

In our experience, the PlantDoc dataset is a valuable resource—it is realistic and diverse, containing information on many different plant species and diseases. In addition, the dataset is well annotated, making it easy to use for developing and testing a variety of models. The dataset described in some detail here comprises a (tomato) subset of the PlantDoc database. **Figure 3** illustrates the class distribution of the PlantDoc dataset in relation to the instances of the training dataset. Upon examination of the diagram, it becomes evident that the dataset encompasses not only the 27 officially mentioned classes but also two additional classes, namely *Potato Leaf* and *Tomato Two Spotted Spider Mites Leaf*. This implies that the total number of classes in the dataset is 29.

In order to create a model that is as realistic as possible and has been developed in relation to practical requirements, only one plant species, i.e., classes of the tomato plant, from the PlantDoc dataset should be considered. The primary rationale for this is that the tomato plant is a crop of significant global importance, with a particularly prominent role in German-speaking regions. Consequently, it is a highly pertinent subject within the context of realistic studies. Secondly, the tomato plant in the PlantDoc dataset contains the most classes within the dataset. This presents the model to be developed with a sufficient and realistic challenge, because differentiating between a healthy plant and a single disease, for example, would not be sufficiently complex to evaluate the requirements for model quality and the dataset.

**Figure 4:**
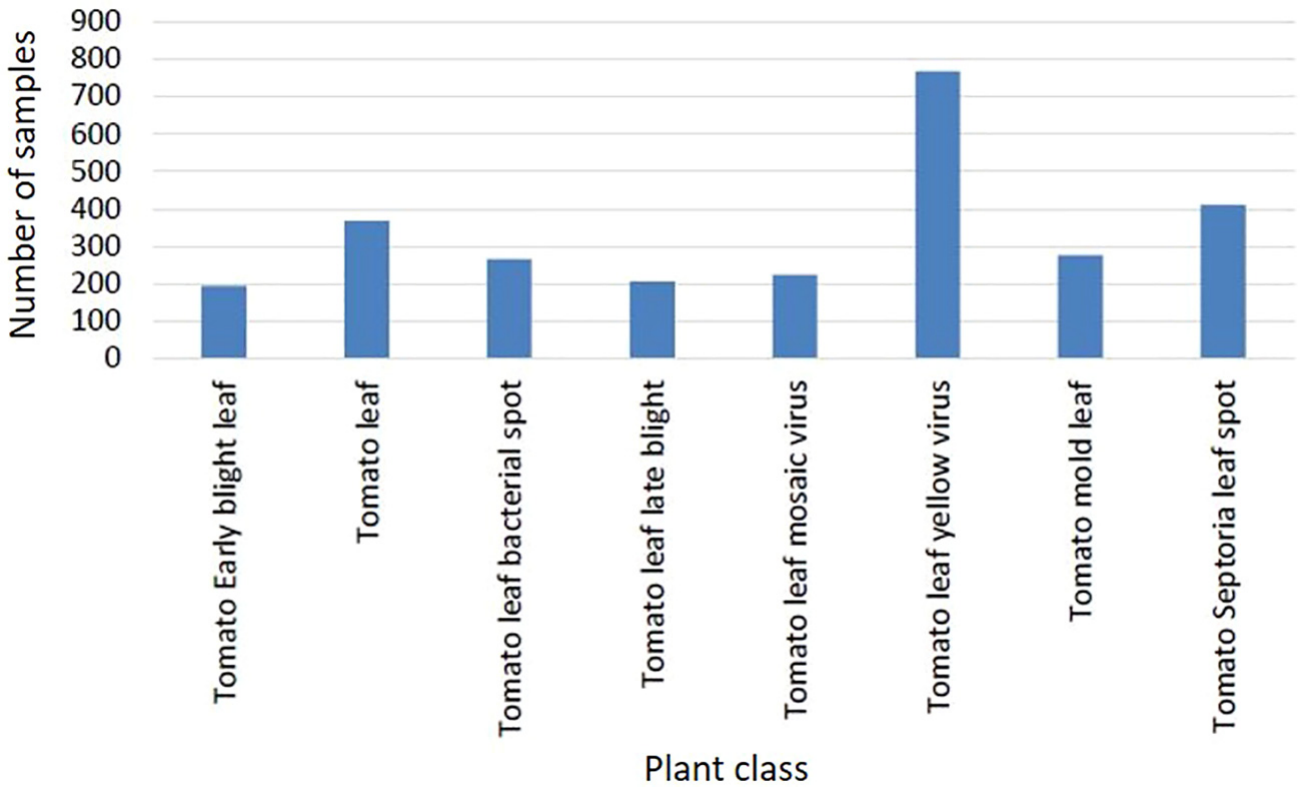
Sample (instance/bounding box) frequencies per disease for the selected tomato dataset extracted from the PlantDoc database.

The class *Tomato Two Spotted Spider Mites Leaf* (see **Figure 3**) contains a very low number of sample images, and is thus excluded here. The resulting dataset comprises 2,753 images from 8 distinct classes, including healthy leaves. **Figure 4** shows the class distribution when considering the tomato plant. The distribution is somewhat unbalanced, but more balanced than for the entire PlantDoc dataset. This imbalance could potentially skew the model towards overrepresented classes, thereby affecting its performance on underrepresented classes.

**Figure 5:**
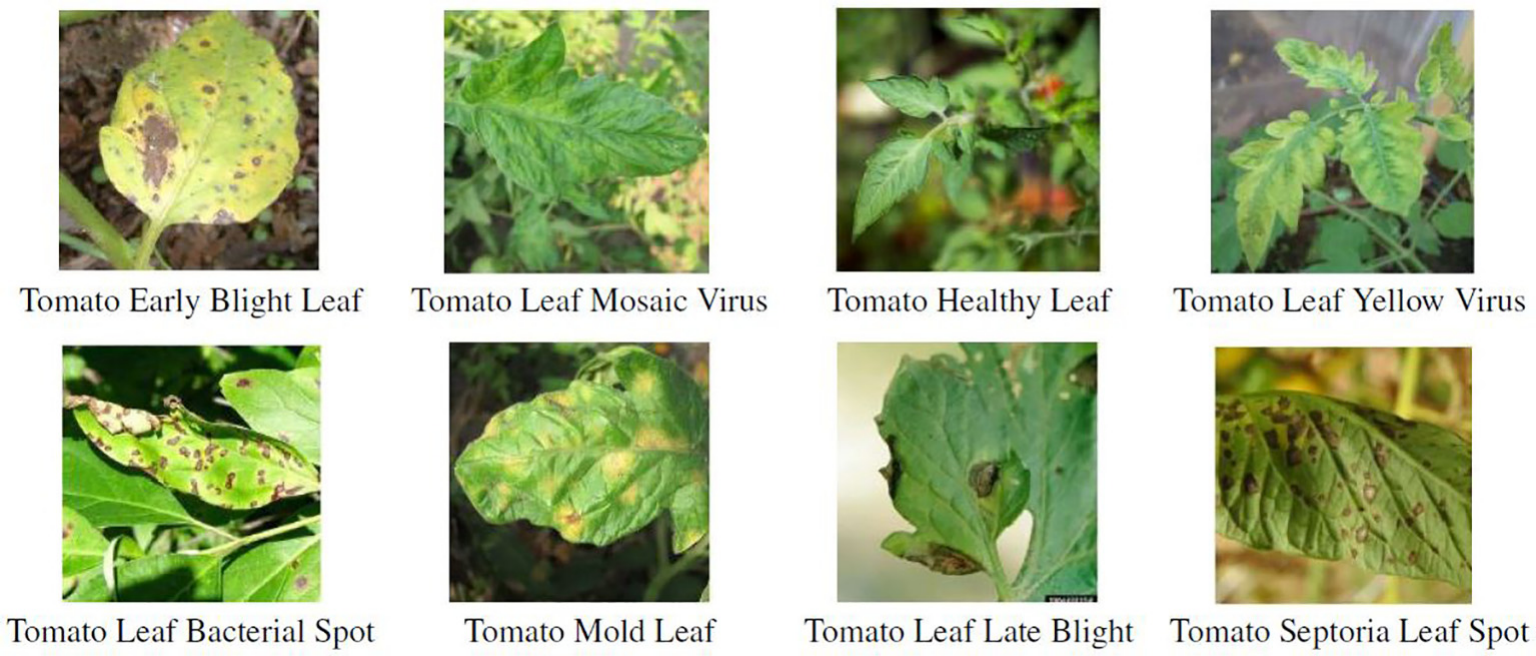
Images of the individual classes of diseases/pests from PlanDoc dataset^4^.

**Figure 6:**
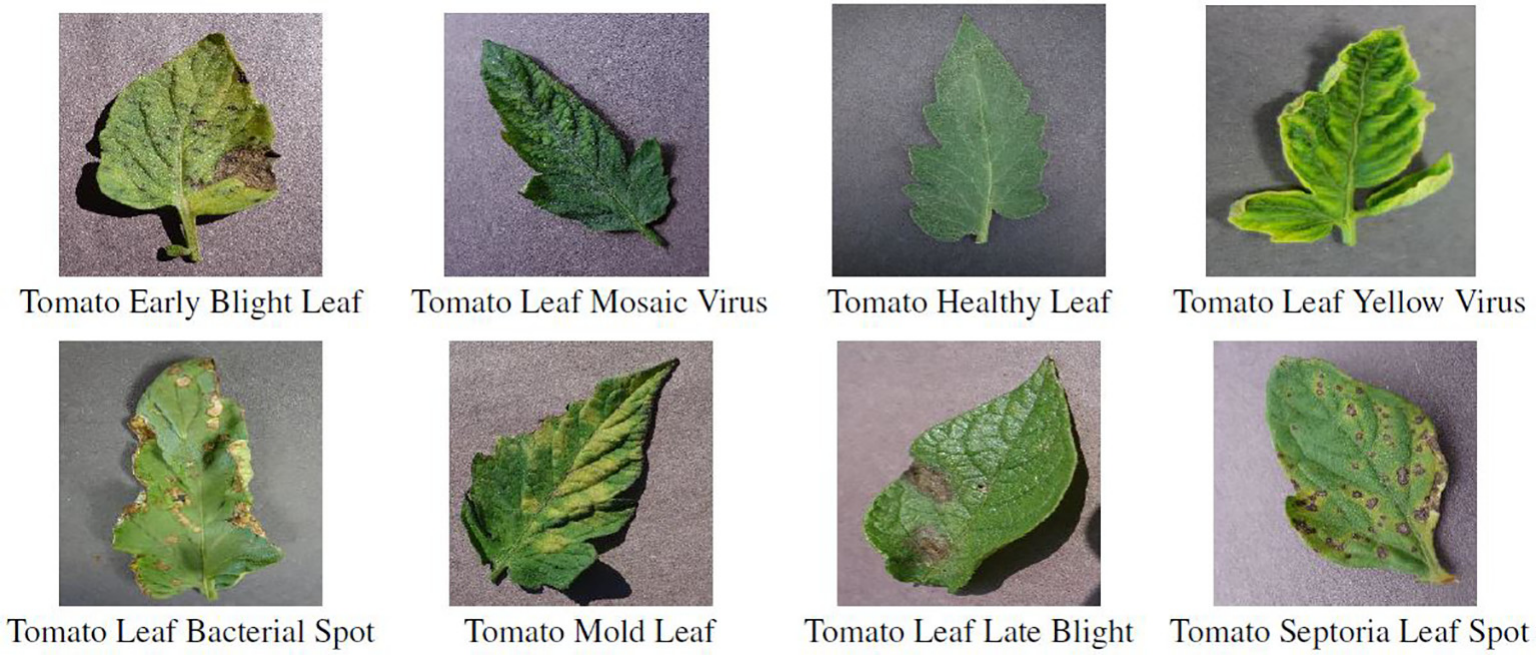
Images of the individual classes of diseases/pests from PlantVillage dataset^5^.

In order to gain a better understanding of the data used and the characteristics of the various disease classes, **Figure 5** shows an example image for each class of the reduced dataset. **Figure 6** contains sample images from PlantVillage dataset for the same diseases as in **Figure 5**, to show the gap between lab-controlled and real-life images. The images serve to confirm the complexity of the various diseases and the similarity of the symptoms. The pictures show healthy leaves and symptoms of four fungal, two viral, and one bacterial disease. With regard to the two viral diseases, only inconspicuous symptoms are displayed, namely, slightly curled leaves. In the absence of sufficient specialist knowledge, such symptoms could be mistaken for those of a healthy tomato plant. The symptoms of fungal and bacterial diseases are consistently manifested as brown or brown-yellow spots of varying sizes on the leaves. While *Tomato Leaf Late Blight* can be readily distinguished from the other diseases by a relatively large spread of brown spots, the symptoms of the other diseases are highly similar. Additionally, *Tomato Mold Leaf* exhibits a somewhat distinct morphology of lesions, which can be employed to differentiate this disease from the others. *Tomato Early Blight Leaf*, *Tomato Septoria Leaf Spot*, and *Tomato Leaf Bacterial Spot* are all typified by small brown lesions distributed across the entire leaf. This can lead to difficulties in differentiating between these diseases, with potential for confusion between fungal and bacterial diseases. In real-field situations, the background for tomato diseases is complicated, and the size of disease spots is small. As weather, lighting, and occlusion affect imaging, disease spot imaging presents a variety of challenges. These include the potential for diverse postures, blurry details in symptom features, high missed warnings, and false alarm rates due to overlapping occlusions (Wang and Liu, 2024).

## 4 Review of the selected papers

The development of novel methodologies for the early detection of plant diseases can markedly enhance the potential for increased agricultural yield. In recent decades, researchers have conducted a multitude of studies aimed at accurately identifying the presence of pathogens in diverse crops. The majority of models demonstrated a high degree of accuracy when typically applied on the PlantVillage dataset or similar databases, containing leaves photographed in a single background, which does not reflect the pose of leaves in the natural environment. However, a significant challenge remains in ensuring their operational efficacy in non-laboratory settings, such as those encountered in agricultural fields or greenhouses. In contrast, some works have employed the PlantDoc dataset or self-acquired datasets, which comprise data pertaining to diseased and healthy plants in authentic farmland settings. For the purposes of this paper, we have limited our investigation to a single plant species, namely the tomato leaf. Furthermore, our research encompasses related works that have proposed methodologies for the detection and classification of plant diseases and pests using images captured in actual field settings, specifically on agricultural premises. In nature, tomato diseases and pests are often obscured by light and shade, and the branches and leaves are covered or overlapping. The identification and localization of tomato diseases and pests under the influence of noise, shadows, overlapping, different textures, etc. in the images is a much more difficult problem than under (ideal) laboratory conditions.

Fuentes et al. (2017) used a deep learning-based framework to detect tomato diseases and pests in real-time, employed three distinct types of detectors, R-CNN, R-FCN), and SSD. A variety of convolutional neural network (CNN) architectures were investigated for potential integration with these detectors, including: The following CNNs were considered: AlexNet, VGG-16, GoogLeNet, ZFNet, ResNet-50, ResNet-101, and ResNetXt-101. It was observed that the application of data augmentation resulted in an increase in mean Average Precision (mAP) of approximately 30%. Moreover, it was discovered that conventional CNN architectures, such as VGG16 and ResNet50, demonstrated superior performance compared to their deeper counterparts, including ResNet-101. However, problems related to class imbalance and false positives remained unresolved. Due to the lack of samples, some classes such as white fly and leaf mold with high pattern variation tended to be confused with others, resulting in false positives or lower average precision.

Subsequent research in (Fuentes et al., 2018) demonstrated enhanced anomaly recognition accuracy by integrating a bank of one-class CNN classifiers to refine the decisions made by the Faster R-CNN. The refinement CNN filter bank effectively mitigated false positives, leading to a notable improvement in mAP of 96.25%. A remaining issue is that the learning process is observed to be more biased towards classes with more samples and variations, due to the limited training data with significantly unbalanced distribution. Moreover, the discrepancy between classes due to inter- and intra-class variations leads to a high number of false positives, which consequently limits the system to achieve higher accuracy in the considered complex recognition framework (Fuentes et al., 2020). To address these problems, an extended version of Fuentes’ approach was then proposed in (Fuentes et al., 2020), which achieved a mAP of 96.25%. The cost-effectiveness of the techniques presented has yet to be demonstrated in large-scale cultivation fields.

Ahmad et al. (2020) used VGG16, VGG19, ResNet, and InceptionV3 and fine-tuned CNNs to obtain optimal results on a tomato leaf dataset containing images of both types, lab-controlled and in-the-wild, and classified 6 disease classes. According to their results, InceptionV3 provided an accuracy of 99.60% on lab-controlled images and 93.70% on field images. Therefore, there is still room to optimize the considered networks for better performance on real field-based data.

Chen et al. (2020) proposed a framework for tomato leaf disease detection, which consists of image denoising and enhancement by Binary Wavelet Transform combined with Retinex (BWTR), removal of noise points and edge points, retention of important texture information, separation of tomato leaves from the background using KSW optimized by Artificial Bee Colony Algorithm (ABCK), and image identification by the Both-channel Residual Attention Network model (B-ARNet). The application results showed that the overall recognition accuracy is about 88.43% based on images taken in natural light using a Nikon camera. However, the collected image dataset is too small and should be further enriched to improve the generalization ability of the model, especially regarding the identification of multiple diseases on the same leaf. Moreover, the preprocessing part of this network is more complex and challenging to implement in a real-time recognition system (Zhang et al., 2023).

Khan and Narvekar (2020) developed a system for classifying tomato plant diseases, including early blight and late blight, using a custom CNN. Their system achieved an overall accuracy of 97.25% using a combined dataset, which was collected from various sources, including PlantVillage, internet data, and real-world images from Tansa Farm captured in an uncontrolled environment. However, this dataset is largely idealized and requires expansion with more diseases. Only two classes of diseases have been considered so far, and the dataset is dominated by images from the PlantVillage dataset, making it more representative of ideal/laboratory conditions than real field scenarios.

In the paper of Liu and Wang (2020), an improved YOLOv3 algorithm was proposed for tomato disease and pest detection. The Yolo V3 network was improved by using multi-scale feature detection based on image pyramid, object bounding box dimension clustering and multi-scale training. The experimental results showed that the detection accuracy of the algorithm was 92.39%. Compared with SSD, Faster R-CNN, and the original YOLOv3, the improved YOLOv3 CNN achieved higher detection accuracy. Although real field condition images were used for training, the images were categorized based on pathogens. The interclass similarity of lesions at different stages can confuse the CNN and thus cause misclassification (Cheng et al., 2022). Moreover, the proposed method can only effectively detect tomato disease and pest targets in the case of slight leaf overlap, and there is no satisfactory detection result in the case of large area occlusion (Wang et al., 2021b).

In (Natarajan et al., 2020), different DL architectures were used to identify pests and diseases: Faster R-CNN, R-FCN and SSD. Based on the tomato pest and disease detection study conducted on field data, the Faster R-CNN deep learning architecture combined with ResNet provided better performance compared to R-FCN and SSD; it achieved a mAP of 80.95%. However, Faster R-CNN as a two-stage object detection method usually requires classification and regression of a large number of candidate regions in the image, as well as separate forward inference for each candidate region, which consumes a lot of computational resources and is not suitable for real-time scenes. Moreover, the images in the used dataset were taken from very specific areas in India, and it is still necessary to add images from other locations.

Ouhami et al. (2020) evaluated the efficacy of deep learning models, specifically DensNet-161, -121, and VGG-16 with transfer learning, for the detection of tomato crop diseases in standard RGB images. The study is based on images of infected plant leaves, which were divided into six categories: pest attacks, plant diseases, and other types of infections. The database of images was developed earlier by El Massi et al. (2016). The results were promising, with an accuracy of up to 95.65% for DensNet-161, 94.93% for DensNet-121, and 90.58% for VGG-16. However, the dataset used is too small and should be expanded with many more real tomato disease samples from different fields to verify or improve the model accuracy.

In their study, Sharma et al. (2020) trained two CNN models on a tomato dataset comprising nine disease types and healthy leaves. The first model, designated F-CNN, employed full leaf images with diverse backgrounds and disease progression, whereas the second model, S-CNN, utilized images segmented to include only regions of interest with disease symptoms. The regions of interest were selected to encompass multiple lesions of a similar disease. In comparison to the F-CNN model trained on full images, the S-CNN model trained on segmented images demonstrated a significant improvement in performance, reaching an accuracy of 98.6% when tested on an independent dataset that had not been previously seen by the models, even with 10 disease classes. However, the proposed detection system had difficulty in cases where regions contained symptoms of multiple diseases. Masking or cropping to isolate single disease symptoms could alleviate this problem to some extent. In addition, the method can be very sensitive to the quality of the segmentation, which depends on the human involved in the segmentation (Sharma et al., 2020). Also, the dataset is dominated by PlantVillage images, which represent controlled conditions with similar backgrounds and lighting.

Fuentes et al. (2021) proposed and explored a paradigm called “control to target classes” to improve the performance of their DL-based detector to deal with changes in new greenhouse conditions using target and control classes. They created a more extended dataset than in (Fuentes et al., 2017, 2018), including more classes and samples, and obtained a recognition rate of 93.37% mAP for the target classes during inference. The main limitation of the proposed method is the data imbalance. This issue has a direct impact on the selection of target classes as the recognition objective of a system. The data should be sufficient to capture all the features that the system may encounter in real greenhouse scenarios (Fuentes et al., 2021).

The objective of the study conducted by Khatoon et al. (2021) was to develop an integrated system capable of diagnosing critical crop issues in real-time with a high degree of accuracy. The researchers employed a variety of DL models to recognize and predict different diseases caused by pathogens, pests, and nutritional deficiencies. A variety of CNNs were trained on a substantial dataset comprising images of tomato leaves and fruits. The performance of two distinct network architectures was evaluated: ShallowNet, a shallow network trained from scratch, and a state-of-the-art deep learning network, which was fine-tuned via transfer learning. In their experiments, DenseNet demonstrated consistent high performance, achieving an accuracy score of 95.31% on the test dataset. However, the dataset used was not large enough and showed imbalances.

Wang et al. (2021a) proposed an improved YOLOv3-tiny method for real-time detection of tomato diseases and pests under occlusion and overlap conditions in real natural environment. The results showed that the mAP under three conditions a) deep separation, b) debris occlusion, and c) leaf overlapping are 98.3, 92.1, and 90.2%, respectively. In the latter cases, there is still room to improve the approach and expand the dataset to include more types of plant diseases and pests. YOLOv3 seems to struggle when applied directly to certain specific detection objects in complex scenes with varied features.

In another research, Wang et al. (2021b) enhanced the YOLOv3 model for the early detection of tomato pests and diseases in complex backgrounds, achieving enhanced detection results. The test results of nine common tomato diseases and pests under six different background conditions are statistically analyzed. The proposed method has an F1-score of 94.77% and an mAP value of 91.81%. The test results show that the method is suitable for the early detection of tomato diseases and pests using large-scale video images collected by the Agricultural Internet of Things. However, the model needs to be improved for issues such as small objects and occluded objects that tend to be missed or inaccurate positioning of the detection frame.

For the automatic identification of tomato anomalies in complex natural environments, Wang and Liu (2021) proposed a YOLO variant called YOLO-Dense based on the YOLOv3 framework. They added a dense connection module in the network architecture to improve the network inference speed of the model, and adopted a multiscale training strategy to improve the recognition accuracy of objects at different scales. The experimental results showed that the mAP of the YOLO-Dense network is 96.41%, surpassing SSD, Faster R-CNN, and the original YOLOv3 network. However, the dataset used should be expanded with many more real tomato disease samples from other regions of the world to verify the achieved model accuracy.

In their paper, Ahuja et al. (2022) put forth a model that integrates the ResNet and InceptionNet architectures in a novel approach. The model achieved an accuracy of 99.08% on the PlantVillage dataset and a reasonable accuracy of 66.06% on the PlantDoc dataset. Furthermore, the authors propose a method for enhancing the detection of diseases in crops in real-world scenarios by augmenting the number of data points. They also discussed the potential benefits of deformable convolution, which is capable of learning various geometric transformations and can improve the performance of the architecture. However, the model needs to become more robust and significantly more accurate, especially for the PlantDoc dataset and similar datasets from real or natural fields.

Astani et al. (2022) conducted an in-depth analysis and classification of 13 distinct categories of tomato diseases, utilizing a combination of subsets of the databases Plantvillage (laboratory setting) and Taiwan Tomato leaves (farm setting); see (Huang and Chang, 2020). The Taiwan tomato leaves database comprises images that have been subjected to a number of challenges, including fluctuations in brightness, the presence of multiple leaves, the presence of background clutter, shadows cast upon the leaves, a reduction in image quality, and the display of diverse textures in accordance with the conditions prevailing on farms. To this end, 260 ensemble classifiers were devised, employing diverse preprocessing techniques, distinct feature extraction methodologies, and varying classifiers. The optimal ensemble classifier attained an accuracy of 95.98%. Furthermore, a comparison was conducted between the numerous DL models and the 260 proposed ensemble models within the proposed methodology. The experiments of Astani et al. (2022) showed that CNN accuracy is very sensitive to the similarity of training and test data, and that low accuracy is obtained when the model is trained on lab data, such as PlantVillage, and tested on field data. In this context, the ensemble strategy should be made robust and more accurate on field datasets by creating diversity in classifier models or by changing the number of classifiers.

The work of Cheng et al. (2022) developed a system for automatic identification of eight tomato disease and pest categories using images of tomato leaves collected in the field or greenhouse. The proposed multi-stage system consists of an anomaly detection model (CNN 1), a disease identification model (CNN 2), a leaf mold/mildew II discrimination model (CNN 3), and a chatbot controller. CNN 1 achieved an accuracy of 97.40% in detecting anomalous images, and CNN 2 achieved an accuracy of 93.63% in categorizing images into the considered tomato disease and pest categories. Although high performance was achieved under challenging real field conditions, some unsuccessful cases of lesion detection and disease and pest identification were observed using the test images. Since the varieties studied were large tomatoes, the diseases considered in the dataset (specks, powdery mildew, systemic disorders, etc.) are somewhat special, i.e. different from the traditional ones (spots, blights, molds, etc.) found in other studies. Therefore, the combination of both types of datasets may be useful in future work.

The work of Liu et al. (2022a) proposed and tested an improved YOLOv4 algorithm on a self-generated pest dataset. The average recognition accuracy reached was 95.2%. However, the pest dataset of this study is relatively small and includes only a few pests and should be greatly expanded, especially for tomato diseases. Only then can the performance of the method be definitively determined.

In (Liu et al., 2022b), a neural network named DCCAM-MRNet was developed using the combination of a filtering algorithm (INLM—Integration NonLocal Means) and ResNeXt50 as a backbone network. The results for tomato leaf disease identification showed that the DCCAM-MRNet had an accuracy of 94.3% in identifying tomato leaf diseases. However, the noise reduction and feature retention capabilities of the image denoising algorithm it used need to be improved. A more suitable denoising algorithm for tomato leaf disease images is needed to improve image quality (Zhang et al., 2023).

In their study, Xu et al. (2022) presented a novel data augmentation paradigm, called the style-consistent image translation (SCIT) model, which can adapt variations from one class to another. Extensive experiments were conducted on three tasks, image classification, object detection, and instance segmentation, and suggested that the SCIT algorithm can improve the performance of various deep learning-based methods and outperform the state-of-the-art data augmentation methods. The proposed model obtained a mAP of 68.3%, which is comparable to the values obtained in other studies, but it is far from being satisfactory.

Bellout et al. (2023) employed images of tomato leaves from two datasets, PlantVillage and PlantDoc, and combined them to form a diverse and comprehensive dataset for the purpose of training the models under investigation. A comparative analysis was conducted of four versions of the widely used YOLO (object detection model), specifically YOLOv5, YOLOX, YOLOv7, and YOLOv8. The results demonstrated that YOLOv5 achieved the best level of accuracy among the four versions, with a score of 92.7%. To optimize the model’s hyperparameters, hyperparameter evolution approaches were applied, resulting in a notable enhancement, with the accuracy reaching 93.1%. However, only three different disease categories (Tomato Late Blight, Tomato Septoria Leaf Spot, and Healthy) were used in the study. Therefore, the dataset is not representative of real-world multi-disease scenarios.

With a substantial collection of over 18,160 images pertaining to tomato diseases, the PlantVillage dataset represents one of the most comprehensive and extensively studied public repositories of plant disease data. However, the dataset was created in a laboratory setting and thus may not reflect the nuances of real-world images. Models trained on this dataset tend to perform poorly on real-world images. Some natural or real-world datasets are available, but they are proprietary and not publicly accessible. To address these challenges, Gehlot et al. (2023) proposed the creation of a new publicly available dataset, designated “Tomato-Village,” comprising three variants: (a) multiclass tomato disease classification, (b) multilabel tomato disease classification, and (c) object detection-based tomato disease detection. The three variants of the dataset have been subjected to analysis using a range of convolutional neural network (CNN) architectures and models. All variants of the YOLOv7 and YOLOv8 architectures demonstrated satisfactory performance, with an mAP50 value approaching 90%. The YOLOv7 and YOLOv8-m achieved mAP50 of 98.3% and 96.4%, respectively. YOLOv7 was the best-performing model, but YOLOv8-m also performed quite well. Additionally, YOLOv8-m is smaller in size, requires less training time, and has a faster detection speed (Gehlot et al., 2023). However, the images in the used dataset were taken from very specific open fields in Jaipur and Jodhpur. It is still necessary to add images from other locations, as well as from real or natural environments, such as polyhouses, greenhouses, etc. Only then can the robustness of the approach be finally assessed.

Khan et al. (2023) presented a transformer-based model called TomFormer (Tomato TransFormer) for tomato leaf disease detection. They proposed to use a fusion model combining a ViT and a CNN. TomFormer achieved mAP scores of 87%, 81%, and 83% on the KUTomaDATA (proprietary dataset), PlantDoc, and PlantVillage datasets, respectively. Surprisingly, TomFormer had a relatively lower mAP score on the PlantVillage dataset, contrary to most studies in the literature. This result can be attributed to the specific challenges of the dataset, where TomFormer has difficulty distinguishing between diseases with subtle visual differences due to the uniform background setting Khan et al. (2023).

In the work of Liu and Wang (2023), a tomato disease object detection method (improved YOLOv6) that integrates prior knowledge attention mechanism and multi-scale features, called PKAMMF, was proposed. The experimental results on the home-made tomato disease dataset from real natural environment demonstrated the effectiveness of the proposed approach, and it achieved a mAP of 91.96%, which is an improvement of 3.86% compared to the baseline methods. However, the performance of the proposed method should be verified by applying it to datasets other than those representative of plant diseases in a specific region of the world (in China).

Rajamohanan and Latha (2023) constructed a customized field dataset consisting of multiple images of tomato leaves taken with a mobile phone from agricultural fields in Indian regions and classified into two categories: healthy and diseased. In their study, YOLOv5 was used to classify images of tomato leaves into the respective categories and achieved an accuracy rate of 93% on the test dataset, which was significantly higher compared to the Faster R-CNN and EfficientDet mode ls. However, the dataset used remains small and only representative of plant diseases in a very specific region of the world (in India), and the proposed method should be applied to other or expanded datasets.

One limitation of some CNN models is that they do not perform well with small datasets and fail in cases where samples have symptoms of multiple diseases or viruses in the same image of the dataset. Singh et al. (2023) addressed this issue by using transfer learning with pre-trained models (InceptionV3, VGG-16, and ResNet-50) to improve classification accuracy. The classification accuracy for a dataset comprising 5,500 images was determined to be 86% for a custom CNN, 79% for VGG-16, 91% for ResNet-50, and 92% for InceptionV3. The classification accuracy was found to be at least 6% higher when pre-trained deep learning models were used for transfer learning in comparison to a custom CNN model. However, the CNNs used have had problems with specimens with multiple diseases, which can lead to incorrect training and lower accuracy. Transfer learning with pre-trained models can only mitigate this problem to a certain extent.

An image-based approach for leaf disease detection in tomato using PLPNet, a YOLOX-S-based object detection approach for tomato leaf diseases, was proposed by Tang et al. (2023). The experimental results show that PLPNet achieved a mAP50 of 94.5% and an average recall of 54.4% on a home-grown dataset. The model is more accurate and specific for tomato leaf disease detection than other popular detectors such as Faster R-CNN, RetinaNet and YOLOv4. However, the custom dataset used in this study eliminated several blurry and low-quality images, which aided model training. Therefore, the method needs to be evaluated on other or extended datasets.

A new identification network structure, called Multi-channel Automatic Orientation Recurrent Attention Network (M–AORANet), was designed in the paper (Zhang et al., 2023) by analyzing the characteristics of tomato leaf disease, which has a superior ability in the tomato leaf disease identification task. The approach solves the problem of low accuracy in tomato leaf disease identification caused by image noise and inter-class similarity and intra-class variability in current identification networks in applications. Experiments showed that M–AORANet has a recognition accuracy of 96.47% and an F1-score of 93.96%. However, the model developed had some difficulties in identifying diseased leaves in images that were obscured by other leaves or objects. The same was true for some images in which the diseased leaves are in the early stage of the disease and their characteristics are not yet fully revealed. These two factors made the M–AORANet network perform poorly in the application, and should be solved in future work.

Chen et al. (2024) proposed a dual vision transformer (DVT) classification model that includes densely connected networks and transformer modules. Experiments showed that the proposed model achieved 95.4% accuracy on a newly combined dataset. The paper also proposed a cycle-consistent generative adversarial network (GAN)-based transformer model to generate diseased tomato leaf images for data augmentation. The application on the augmented dataset further improved the accuracy to 97.6%. However, the dataset should be expanded with more complex, diverse, and realistic diseases to meet the needs of real-world applications and improve the generalizability of the model.

Tej et al. (2024) focused on the application of the YOLOv5 algorithm for the simultaneous detection and localization of multiple plant diseases on leaves. They compared YOLOv5s and YOLOv5x models and demonstrated the superior performance of YOLOv5x, which achieved a mAP of 96.5% on a self-generated dataset collected in the Monastir region of Tunisia. However, as with the study by Rajamohanan and Latha (2023), the dataset used remains small and only representative of plant diseases in a specific region of the world (in Tunisia). In addition, the proposed method should be further evaluated under more complex conditions with multiple leaves and occlusions.

Umar et al. (2024) proposed a target detection model based on an improved version of YOLOv7 to accurately detect and categorize tomato leaves under harsh field conditions. The university greenhouse served as the source for a data sample collected from the leaves of tomato plants. The study achieved an accuracy rate of 98.8%. To facilitate immediate disease detection in remote agricultural areas, the model framework and computational requirements must be optimized for placement in resource-constrained environments, such as those limited by mobile devices.

In their study, Wang et al. (2024) proposed a method for detecting diseases on tomato leaves based on the fusion of attentional mechanisms and multiscale features. Their approach achieved an average accuracy of 92.9% in the detection of tomato leaf diseases. However, the method is not effective in dealing with small disease spots with similar symptoms under complex backgrounds. Under conditions of light and shadow interference, the results (for YOLOX and YOLOv6-s) also revealed some false positives and false negatives, such as misidentifying late blight as early blight. YOLOv5, YOLOX, YOLOv7, and YOLOv8 showed poor performance in the handling of occlusions that are present in the images. Therefore, the proposed model structures still have room for further improvement, especially when applied to the public FieldPlant dataset.

Wang and Liu (2024) introduced a real-time tomato disease detection algorithm using Swin-DDETR, Meta-ACON and IBiFPN, named TomatoDet, to improve the performance by optimizing the backbone network, activation functions, and feature fusion structure. Its effectiveness has been demonstrated by experimental results, which show improved detection accuracy for tomato diseases. It outperforms conventional disease detection algorithms, achieving a mAP of 92.3% on a home-made tomato disease dataset. However, the dataset used is too small and should be expanded with many more real tomato disease samples from different fields to verify the achieved model accuracy.

Zhong (2024) improved four types of loss functions and replaced the backbone network of YOLOv8 to make the model more accurate. The new YOLOv8 version (with FasterNet as the backbone) had an mAP of 84.5%. However, this performance level is still moderate and the model structure should be improved to achieve higher accuracy in tomato disease detection.

## 5 Studies analysis and discussion

**Figure 7:**
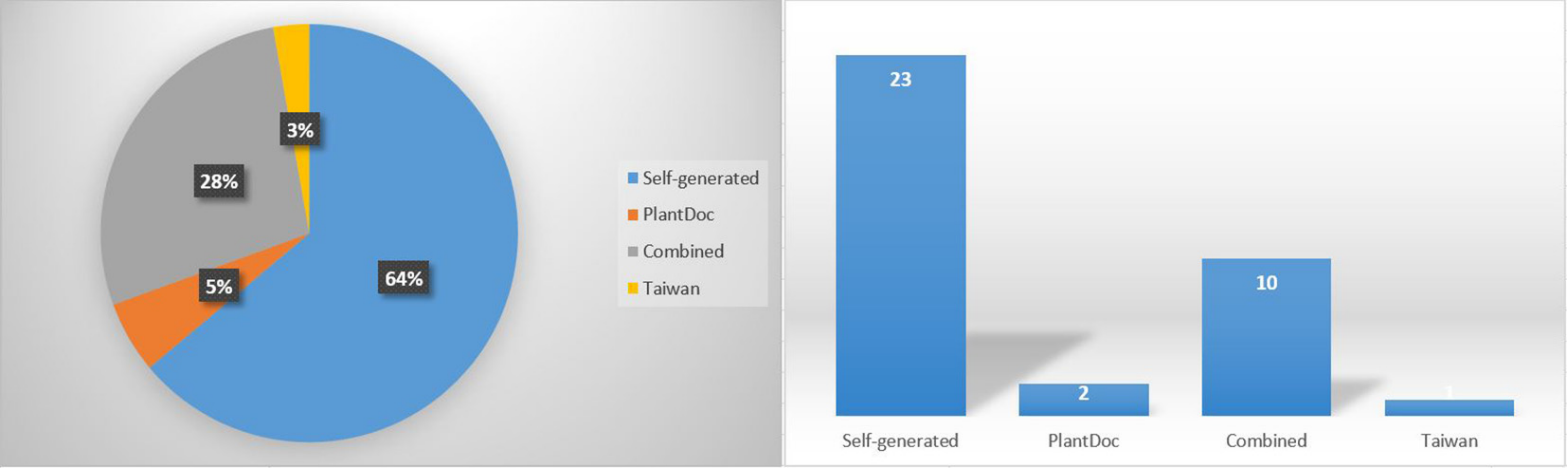
Sources of used datasets.

The results given in **Table 2** show that the performance differs significantly across the benchmark datasets and the CNN networks. **Figure 7** shows the distribution of the reviewed studies across the four main categories of dataset sources (Self-generated, PlantDoc, Combined, and Taiwan) considered in the studies. Note again that the PlantVillage dataset is only relevant here for studies that used it in combination with other datasets, i.e. in the “Combined” category. The largest dataset used in this context was that constructed by Khatoon et al. (2021). It can be seen that self-generated datasets are the most common source of data. The largest self-generated dataset from a real natural environment was that of Wang and Liu (2021). Combined datasets rank second. Despite being a valuable resource, the PlantDoc dataset is surprisingly underutilized.

**Table 2:** Comparative analysis of existing methods for the recognition of tomato leaf diseases, utilizing at least partially real-field datasets.

Ref. (Year)	Data source (dataset size: # of images) – Disease/pest classes	Performance metric: CNN architecture—value	
Fuentes et al. (2017)	Self-acquired images on farms located in Korea (**5,000**) – **10** classes: Leaf Mold, Gray Mold, Canker, Plague, Miner, Low Temperature, Powdery Mildew, Whitefly, Nutritional Excess, Background	mAP: Faster R-CNN with VGG-16/ResNet-50/ ResNeXt-50—83.0/75.37%/71.1%, SSD with ResNet-50—82.53%, R-FCN with ResNet-50—85.98%	
Fuentes et al. (2018)	Self-acquired images (**8,927**) – **10** classes: Leaf Mold, Gray Mold, Canker, Plague, Miner, Low Temperature, Powdery Mildew, Whitefly, Yellow Leaf Curl, Nutritional Excess	mAP: Faster R-CNN with VGG16 feature extractor—96%	
Ahmad et al. (2020)	Self-acquired data from various fields in a natural uncontrolled environment (**317**) – **6** classes: Healthy, Late Blight, Septoria Leaf Spot, Yellow-Curved	Accuracy: VGG16—84.10%, VGG-19—86.30%, ResNet—91.30%, InceptionV3—93.70%	
Chen et al. (2020)	Self-acquired data at the demonstration base of Hunan Vegetable Institute, Changsha, China (**2,716**) – **5** classes: Early Blight, Late Blight, Citrinitas Leaf Curl, Leaf Mold, Bacterial Leaf Spot	Accuracy: AlexNet—83.62%, ResNet-50—84.16%, ARNet—85.12%, B-ARNet—88.43%	
Fuentes et al. (2020)	Self-acquired images in real-field scenarios (**8,927**) – **11** classes: Leaf Mold, Gray Mold, Canker, Plague, Miner, Low Temperature, Powdery Mildew, Whitefly, Nutritional Excess, Yellow Leaf Curl, Background	mAP: Faster R-CNN with VGG-16—82.55%, Refinement Filter Bank—96.25%	
Khan and Narvekar (2020)	Subset of PlantVillage (**9,000**) + Internet data (**4,298**) + data from Tansa Farm (**250**) – **3** classes: Early Blight, Late Blight, Healthy	Accuracy: Custom CNN—97.25%	
Liu and Wang (2020)	Self-built dataset from real natural environment (**15,000**) – **12** classes: Early Blight, Late Blight, Yellow Leaf Curl Virus, Brown Spot, Coal Pollution, Gray Mold, Leaf Mold, Navel Rot, Leaf Curl Disease, Mosaic, Leaf Miner, Greenhouse Whitefly	Accuracy: Improved YOLOv3—92.39%, YOLOv3—88.31%, SSD—84.32%, Faster R-CNN—90.67%	
Natarajan et al. (2020)	Self-acquired images in tomato farms in Kallur, Mangapuram, and Piller in Andhra Pradesh, India (**1,090**) – **5** classes: Early Blight, Leaf Curl, Septoria Leaf Spot, Healthy, Bacterial Spot at Early	mAP: Faster R-CNN—80.95%	
Ouhami et al. (2020)	Self-acquired images in farms in the area of Sous Massa, Morocco, extended with images collected from Internet (**666**) – **6** classes: Early Blight, Late Blight, Powdery Mildew, Leaf Miner Flies, Thrips, Tuta Absoluta	Accuracy: DenseNet-161—95.65%, DenseNet-121—94.93%, VGG-16—90.58%	
Sharma et al. (2020)	Subset of PlantVillage (**16,579**) + Internet images (**637**) – **10** classes: Bacterial Spot, Early Blight, Healthy, Late Blight, Leaf Mold, Septoria Leaf Spot, Spider Mite, Target Spot, Tomato Mosaic Virus, Yellow Leaf Curl Virus	Accuracy: Custom F-CNN—42.3%, S-CNN—98.6%	
Fuentes et al. (2021)	Self-acquired images on farms located in Korea (**1,981**) – **5** classes: Leaf Mold, Canker, Gray Mold, Yellow Leaf Curl Virus, Powdery Mildew	mAP: VGG-16—87.06%, ResNet-50—87.34%, ResNet-50 FPN—89.78%	
Khatoon et al. (2021)	Subset of PlantVillage, extended with data from agriculture farms of King Faisal University, Alahsa, Saudi Arabia, under natural conditions (**23,716**) – **24** classes: Mosaic Virus, Bacterial Spots, Yellow Leaf Curl Virus, Late Blight, Early Blight, Leaf Mould, Septoria Leaf Spot, Target Spot, High Temperature, Powdery Mildew, Leaf Miner, Two-spotted Spider Mite, Whitefly, Melon Fly, Melon Thrips, Green Peach Aphid, Taro Caterpillar, Beet Armyworm, Cotton Bowl Worm, Nitrogen Deficiency, Potassium Deficiency, Calcium Deficiency, Magnesium Deficiency, Tomato Healthy	Accuracy: ShallowNet-8—78.05%, VGGNet-16—80.32%, ResNet-50—92.01%, ResNet-152—90.85%, DenseNet-121—95.31%	
Wang and Liu (2021)	Self-built dataset from real natural environment (**15,000**) – **12** classes: Early Blight, Late Blight, Yellow Leaf Curl Virus, Brown Spot, Coal Pollution, Gray Mold, Leaf Mold, Navel Rot, Leaf Curl Disease, Mosaic, Leaf Miner, Greenhouse Whitefly	mAP: YOLOv3-Dense—96.41%, YOLOv3—88.31%, SSD—84.32%, Faster R-CNN—90.67%	
Wang et al. (2021a)	Self-acquired dataset from an experimental tomato planting base in Shouguang City, Shandong Province, China (**5,000**) – **12** classes: Early Blight, Late Blight, Yellow Leaf Curl Virus, Brown Spot, Coal Pollution, Gray Mold, Leaf Mold, Navel Rot, Leaf Curl Disease, Mosaic, Leaf Miner, Greenhouse Whitefly	mAP: Improved YOLOv3-tiny—93.1%, R-CNN—86.6%, Mask R-CNN—87.1%, SSD—85.3%, YOLOv3—88.8%, YOLOv3-tiny—88.1%	
Wang et al. (2021b)	Self-acquired dataset/videos from an experimental tomato planting base in Shouguang City, Shandong Province, China (**10,696**) – **12** classes: Early Blight, Late Blight, Powdery Mildew, Spot Blight, Gray Mold, Leaf Mold, Gray Leaf Spot, Leaf Miner, Whitefly	F1-score: Improved YOLOv3—94.77%, YOLOv3—91.43%, Faster R-CNN—89.04%, SSD—88.45%	
Ahuja et al. (2022)	Subset of PlantDoc (**741**) – **8** classes: Leaf Mold, Bacterial Spot, Septoria Spot, Early Blight, Late Blight, Yellow Virus, Mosaic Virus, Healthy	Accuracy: ResNet-50—26.5%, InceptionResNet—66.0%	
Astani et al. (2022)	10 classes of Plantvillage (laboratory conditions) + 6 classes of Taiwan tomato leaves (farm conditions) – Bacterial Spot, Early Blight, Healthy, Late Blight, Leaf Mold, Septoria Leaf Spot, Spider Mites, Target Spot, Mosaic Virus, Yellow Leaf Curl Virus, Powdery Mildew, Gray Spot, Black Mold	Accuracy: Different ensemble classifiers (ML models)—84.80–86.95%, DenseNet-169—93.66%, EfficientNet-B4—92.83%, Xception—93.81%, InceptionResNetV2—91.98%	
Cheng et al. (2022)	Images collected in fields or greenhouses at Taiwan Agricultural Research Institute (**8,770**) – **8** classes: Early Blight Early, Bacterial Spot Early, Target Spot Early, Gray Leaf Spot, Bacterial Spot Late, Powdery Mildew I, Early Blight Late, Target Spot Late, Leaf Mold, Powdery mildew II, Late Blight, Leaf Miner, Tomato Chlorosis Virus, Tomato Yellow, Leaf Curl Virus	Accuracy: YOLOv4—93.63%	
Liu et al. (2022a)	Pest images collected in the tomato greenhouse in Shouguang (Shandong, China) (**2,893**) – **4** classes: Whitefly, Aphid, Leafminer, Other	mAP: Improved YOLOv4—93.4%, YOLOv4—87.1%, YOLOv3—73.6%, SSD—72.3%, Faster R-CNN—68.7%	
Liu et al. (2022b)	Dataset from the Hunan Academy of Agricultural Sciences (Changsha, China) demonstration base, compiled using data from tomato greenhouses and the Internet (**2,731**) – **6** classes: Leaf Mold, Septoria Leaf Spot, Yellow Leaf Curl Virus, Mosaic Virus, Target Spot, Two-spotted Spider Mite	Accuracy: ResNeXt-50—85.6%, ResNeXt-50-CA—90.2%, DCCAM-MRNet—94.3%	
Xu et al. (2022)	Images collected from different real farms (**999**) – **6** classes: Healthy, Powdery Mildew, Canker, Leaf Mold, Tomato Chlorosis Virus, Magnesium Deficiency	mAP: Faster R-CNN—51.5%, Mask R-CNN—56.6%, YOLOv3—32.6%	
Bellout et al. (2023)	Subset of PlantVillage (**3,000**) + subset of PlantDoc (**325**) – **3** classes: Septoria Leaf Spot, Late Blight, Healthy	Accuracy (training): YOLOv5s—92,7%, YOLOXs—89,3%, YOLOv7-tiny—87,6%, YOLOv8s—92,2%	
Gehlot et al. (2023)	Tomato-Village/variant(c)–object detection-based dataset from the field in the Jodhpur and Jaipur districts of Rajasthan, India (**1,796**) – **8** classes: Early Blight, Healthy, Late Blight, Leaf Miner, Magnesium Deficiency, Nitrogen Deficiency, Potassium Deficiency, Spotted Wilt Virus	mAP: YOLOv8-n—87.2%, YOLOv8-s—93.1%, YOLOv8-m—96.4%, YOLOv7—98.3%, YOLOv7-tiny—91.3%
Khan et al. (2023)	KUTomaDATA: Images captured within greenhouses in Al Ajban, Abu Dhabi, United Arab Emirates (**939**) – **8** classes: Healthy, Bacterial Spots, Early Blight, Late Blight, Leaf Mold, Septoria Leaf Spot, Mosaic Virus, Yellow Leaf Curl	mAP: TomFormer—87%, YOLOS—80%, DETR—82%, ViT—73%, SwinTransformer—78%
Khan et al. (2023)	Subset of PlantDoc (**700**) – **8** classes)	mAP: TomFormer—81%, YOLOS–77%, DETR—79%, ViT—71%, SwinTransformer—76%
Liu and Wang (2023)	Self-acquired dataset from the tomato planting base in Shouguang City, Shandong Province, China (**10,000**) – **10** classes: Early Blight, Late Blight, Bacterial Spot, Gray Leaf Spot, Gray Mold, Leaf Mold, Yellow Leaf Curl Virus, Mosaic Virus, Canker, Anthracnose	mAP: PKAMMF ((improved YOLOv6))—91.96%, Faster-R-CNN—70.73%, SSD—72.54%, YOLOv3—78.62%, YOLOv4—80.37%, YOLOv5—88.98%, YOLOv7—88.10%
Rajamohanan and Latha (2023)	Images collected from the farm of the Department of Agriculture, Karunya Institute of Technology and Sciences in Coimbatore, TamilNadu and Deesan Farm in Palakkad, Kerala, India (**2,311**) – **2** classes: Healthy, Diseased)	mAP: EfficientDet—35%, Faster R-CNN—45%, YOLOv5—93%
Singh et al. (2023)	Subset of PlantVillage + Internet images (**5,500**) – **9** classes: Bacterial Spot, Early Blight, Healthy, Late Blight, Leaf Mold, Septoria Leaf Spot, Spider Mite, Target Spot, Tomato Mosaic Virus, Yellow Leaf Curl Virus	Accuracy: Cutom CNN—86%, ResNet-50—79%, VGG-16—91%, InceptionV3—92%
Tang et al. (2023)	Subset of PlantVillage (filtered images) (**3,524**) + self-acquired images from Internet (**1,909**) – **6** classes: Late Blight, Bacterial Spot, Septoria Leaf Spot, Leaf Mold, Early Blight, Healthy	mAP50: Faster R-CNN—72.6%, YOLOX-s—86.8%, PLPNet—94.5%, RetinaNet—73.5%, YOLOv4—79.9%, YOLOv5-s—84.7%
Zhang et al. (2023)	Self-acquired images at the experimental tomato base of the Hunan Institute of Plant Protection, Changsha, China (**1,850**) + subset of PlantVillage (**1,273**) – **7** classes: Bacterial Spot, Early Blight, Leaf Mold, Septoria Leaf Spot, Yellow Leaf Curl Virus, Healthy	Accuracy: M–AORANet—96.47%, ResNet-50—87.20%, MobileNetV3—91.23%, ViT—92.46%, SwinTransformer—92.78%, B-ARNet—89.83%
Chen et al. (2024)	Images collected online + images from a real greenhouse (**3,820**) – **8** classes: Bacterial Spot, Early Blight, Late Blight, Leaf Mold, Mosaic Virus, Healthy, Spectoria Leaf Spot, Yellow Leaf Curl Virus	Accuracy: DVT—95.4% (original), 96.5% (with augmentation using cyclic GAN)
Tej et al. (2024)	Self-generated dataset collected from greenhouses located in Bkalta in the region of Monastir, Tunisia (**500**) – **6** classes: Healthy, Leaf Miner, Oidium, Nutrient Deficiency, Mildew, Yellow Curve Virus	mAP: YOLOv5s—94.8%, YOLOv5x—97.6%
Umar et al. (2024)	Self-acquired dataset from the University Plant Nursery (**10,337**) – **8** classes: Bacterial Spot, Early Blight, Late Blight, Leaf Mold, Mosaic Virus, Septoria Leaf Spot, Yellow Curl Virus, Healthy	Accuracy: CNN/Improved YOLOv7—98.8%
Wang and Liu (2024)	Self-acquired dataset from a tomato cultivation facility in Shouguang City, Shandong Province, China (**2,000**) – **5** classes: Late Blight, Gray Leaf Spot, Brown Rot, Leaf Mold, Healthy	mAP: Custom DCNN (Transformer + YOLOv8n)—79.3%, Faster R-CNN—79.8%, YOLOXs—80.9%, YOLOv5s—81.7%, YOLOv7-tiny—83.6%, YOLOv8n—92.3%
Wang et al. (2024)	Subset of tomato dataset on the roboflow1 platform (Bryan, 2023) (**4,659**) – **6** classes: Healthy, Leaf Mold, Leaf Miner, Early Blight, Late Blight, Septoria Leaf Spot	mAP: Improved YOLOv6—93.8%, YOLOX—90.5%, YOLOv5—85.6%, YOLOv6—91.1%, YOLOv6-s—89.1%, YOLOv7—88.6%, YOLOv8—89.8%
Zhong (2024)	Dataset collected from tomato leaves in a real environment (**3,362**) – **10** classes: Healthy, Early Blight, Late Blight, Leaf Miner, Leaf Mold, Mosaic Virus, Septoria, Spider Mites, Yellow Leaf, Curl Virus	mAP50: YOLOv8n—84.3%, YOLOv8 with FasterNet as backbone—84.50%,with HGNetV2—80.4%, with GhostNet—81.0%

**Figure 8:**
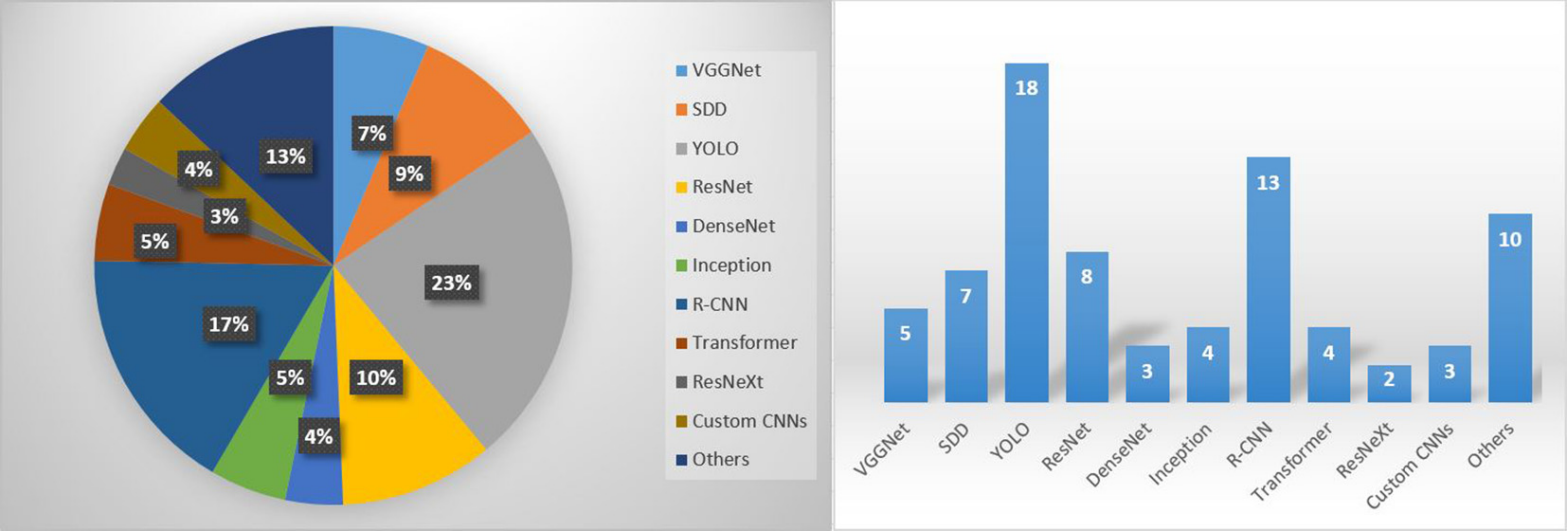
CNN architectures applied in the reviewed studies.

**Figure 8** shows the distribution and number of studies categorized by the CNN architecture used in the field of disease recognition of tomato leaf diseases, utilizing at least partially real-field datasets. Among the summarized studies, YOLO (v3 to v8) was the most widely used architecture. YOLO models can strike a balance between computational complexity and recognition accuracy to some extent (Sajitha et al., 2024), and are therefore well suited for early detection of tomato leaf diseases. The next most commonly used CNNs belong to the R-CNN family (R-CNN, Faster R-CNN, Mask R-CNN). SDD was also employed in many studies. It seems that ResNet is still often considered the baseline. Transformer models are on the rise and their use will probably increase even more in the future. Transformer networks are well suited to the task of feature extraction because they can discover long-range dependencies in data sequences. This is important in tomato leaf disease detection, where symptoms of different diseases often appear in different parts of the leaf (Khan et al., 2023). Thus, transformers are combined with CNNs to perform the entire detection task.

**Figure 9:**
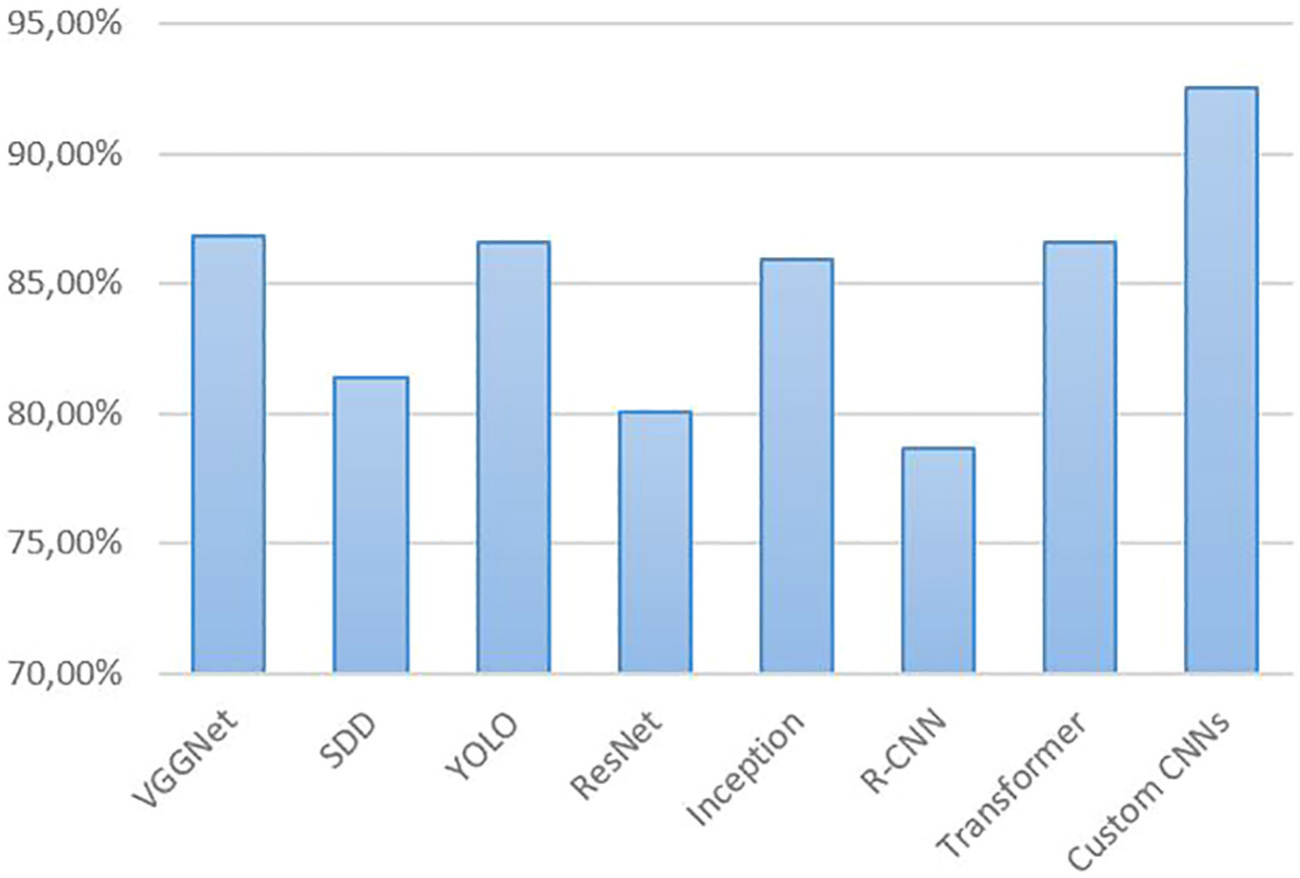
Comparison of the performance (accuracy) of CNN models for tomato disease/pest detection from the results reported in the reviewed studies.

**Figure 9** shows the *average performance* of CNN models used for tomato disease and pest detection in the reviewed studies. The average performance across all models and all papers is 84.66%. Taking just the 7 papers that used PlantVillage subsets, the average performance was 90.18%. Even with self-generated datasets from real agricultural fields, high performance values can be exemplary achieved, i.e., values above 90%. This was the case for Faster R-CNN (Fuentes et al., 2018, 2020) with 96%, InceptionV3 (Ahmad et al., 2020) with 93.70%, DenseNet (Ouhami et al., 2020) with 94.93%, and Xception (Astani et al., 2022) with 93.81%. It is well known that (Faster) R-CNN-based methods can be very accurate, but are not suitable for real-time detection. However, the high accuracy of R-CNN-based models cannot be confirmed here for tomato disease detection using at least partially real field datasets. The average performance of these models across all studies was 78.63% (see **Figure 9**). The RCNN series of algorithms typically require classification and regression of a large number of candidate regions in the image, as well as separate forward inference for each candidate region, which consumes a lot of computational resources and is not suitable for real-time scenes (Zhong, 2024). However, tomato diseases and pests should be detected at an early stage, i.e. in real time, which is important to avoid or reduce the economic loss caused by the disease or pest.

The best CNN architectures and more on the rise are the YOLO variants due their high accuracy and real-time performance. Metrics above 90% have been achieved for improved YOLOv3 by Liu and Wang (2020); Wang et al. (2021b), YOLOv3-Dense by Wang and Liu (2021), improved YOLOv3-tiny by Wang et al. (2021a), YOLOv4 by Cheng et al. (2022), SE-YOLOv5 by Qi et al. (2022), YOLOv5 by Rajamohanan and Latha (2023), YOLOv5s and YOLOv5x by Tej et al. (2024), improved YOLOv6 and YOLOX by Wang et al. (2024), improved YOLOv7 by Umar et al. (2024), YOLOv5s and YOLOv8s by Bellout et al. (2023), and YOLOv8n by Wang and Liu (2024). On average, YOLO models achieved performance values of 86.59% in all the studies considered. A similarly good average performance was achieved by VGGNet with 86.83%. However, the VGGNet architecture has a large number of parameters, which makes it computationally expensive and memory intensive (Singh et al., 2023). As mentioned above, the transformer models are becoming more popular. They also deliver high performance values, with an average of 86.56%. The highest performance (92.58% on average) was achieved by custom CNNs in the studies by (Khan and Narvekar, 2020), Singh et al. (2023), and Tang et al. (2023). However, datasets dominated by PlantVillage data were used in all three studies.

Our analysis can confirm that it is considerably more challenging for CNN models to identify diseases in raw field images than in other forms of data. As expected, when the training and test sets are identical, the presence of extraneous visual elements, such as noise backgrounds and an abundance of foliage in the raw images, impairs the models’ capacity to accurately assess their performance, as stated by (Moupojou et al., 2023). In our comparison, this can be concluded from the results of the studies by Khan and Narvekar (2020) with 97.25%, Sharma et al. (2020) with 98.6%, Khatoon et al. (2021) with 95.31%, and Astani et al. (2022) with 93.81%. These studies used combined datasets dominated by a subset of PlantVillage.

The discrepancy between the results obtained with PlantVillage and those obtained with PlantDoc can be seen in (Ahuja et al., 2022) and (Khan et al., 2023). This is not surprising, because the images in PlantDoc are more complex with noisy backgrounds and there can be multiple leaves in one image (Yao et al., 2024). In contrast, PlantVillage uses images collected in a laboratory setting, so it does not address conditions that occur in actual agricultural scenarios (Fuentes et al., 2017). The discrepancy between the outcomes observed with PlantVillage and those obtained with PlantDoc has also been documented by Ahmad et al. (2023a) (for corn disease identification) and Balafas et al. (2023) (for simultaneous identification of species and diseases). In a recent study, Yao et al. (2024) examined the efficacy of diverse CNN backbones and learning methodologies, conducting a comprehensive experiment on the three benchmark datasets: PlantVillage, Plant Leaves, and PlantDoc. The accuracy and F1-scores were above 80% for PlantVillage and Plant Leaves, with many values exceeding 90%. In contrast, the values for PlantDoc ranged from 40% to 50%.

In the study by Moupojou et al. (2023), a series of revealing experiments were conducted on three datasets, namely PlantVillage, PlantDoc, and FieldPlant, with the objective of evaluating the performance of several cutting-edge CNNs (YOLOv8, SSD MobilenetV2, and Faster R-CCN-InceptionResNet) in identifying leaves and diseases from both raw images and cropped images. The latter were obtained by cropping the initial annotated images using bounding box information. The following observations and conclusions were derived from the aforementioned experiments (Moupojou et al., 2023; Mashamba et al., 2024; Chen et al., 2024):

- The accuracy of the models was diminished when they are trained on PlantVillage and tested on PlantDoc or FieldPlant, due to the considerable discrepancy in the structure between the training and test datasets.
- The outcomes yielded by FieldPlant were markedly superior to those obtained by PlantDoc. This discrepancy may be attributed to the fact that FieldPlant contains a more extensive dataset for model training. It is also possible that the inclusion of both field and laboratory images in PlantDoc may had influenced the results.
- Experiments conducted on the cropped images demonstrated that the models exhibited optimal performance when trained and tested on PlantVillage, which featured a single leaf per image with a uniform background. The efficacy of the models was diminished when they were trained and tested on Cropped FieldPlant or Cropped PlantDoc data. Additionally, the complex backgrounds of the cropped images contribute to this discrepancy, as they are not always consistent between two images, even when they identify the same disease. The results were inferior when the models were trained on PlantVillage and tested on Cropped FieldPlant or Cropped PlantDoc, due to the significant discrepancy in image structure. The models are unable to produce accurate results due to the presence of background noise and the inclusion of scrap leaves in the images.
- Experiments in object detection on the PlantDoc and FieldPlant datasets demonstrated that PlantDoc exhibits superior performance in identifying individual leaves from raw images collected in the field. The presence of some laboratory images with uniform backgrounds in the PlantDoc dataset appears to significantly enhance its performance in object detection. Conversely, some plant leaves are only partially visible in certain images of FieldPlant, which could potentially have a detrimental impact on object recognition and detection tasks.
- For all categories of datasets that are unbalanced and have insufficient sample sizes, data augmentation by synthesizing new tomato disease images improves the robustness and generalization of the classification models.

These guidelines offer valuable insight into the selection of an appropriate database. However, it should be noted that the findings of the study by (Moupojou et al., 2023) were derived from a multi-plant context, which is not within the scope of the review presented in Section 4. The same applies to Yao et al. (2024) who investigated the effectiveness of different backbone CNNs and learning approaches for plant identification and disease classification using “mixed” plant-leaf images as input data.

Given that tomato diseases and pests can vary in different regions of the world due to factors such as shape, variety and environmental conditions, most of the real field or natural environment datasets used in the reviewed studies contain only a limited number of categories of local or regional plant diseases and pests. Therefore, there is still a great need to compile much larger databases, including diseases and pests from different regions of the world, and to apply DL approaches to such diversified datasets.

## 6 Conclusions

Agriculture is suffering from a number of problems; plant diseases and pests are contributing as the most devastating factor. Diseases on the leaves of tomato plants have a negative impact on both quality and yield. Deep learning has shown great potential in improving tomato disease and pest detection, offering high accuracy and efficiency compared to traditional methods. A large number of studies have shown that CNN models have been successfully applied to tomato plant disease and pest detection, with excellent performance on ideal/laboratory datasets.

However, it still needs further development before it can be considered a reliable and widely applicable technology for use in real-world scenarios. To the best of our knowledge, this is the first review, which covers only those studies available in the literature that used data from real agricultural fields. It has been shown that there are only a relatively small number of such studies. With some self-generated datasets from real agricultural fields, high performance values above 90% can be achieved by applying different (improved) CNN architectures such as Faster R-CNN and YOLO. For real-time detection, the YOLO Series algorithms are the preferred methods due to their excellent balance between speed and accuracy. Given these common yet challenging problems in complex natural environments, there is still a need to improve the accuracy of existing methods for detecting tomato leaf diseases. In addition, most of the performance values are lower than those obtained using datasets collected in a laboratory setting such as PlantVillage. However, many of the natural or real-world datasets found in this review are still private and not publicly available.

Future research should address these challenges by developing more robust, explainable, and field deployable models. In subsequent research, we intend to investigate a larger number of classification and object detection algorithms on a wider range of datasets, including diverse diseases and pests from different regions of the world. In the medium term, the integration of these models with IoT and edge computing platforms holds promise to transform agricultural practices, leading to more sustainable and efficient crop management. This requires models that are not only accurate but also lightweight and computationally efficient.

## Footnotes

1 Source of photos: https://pixabay.com/de/photos/tomaten-pflanze-gem%C3%BCse-lebensmittel-1026096/.

2 Source of photos: https://balkongarten-blog.de/tomatenkrankheiten/.

3 Source of photos: https://plantpath.ifas.ufl.edu/u-scout/tomato/early-blight.html.

4 Source of photos: https://www.kaggle.com/datasets/abdulhasibuddin/plant-doc-dataset.

5 Source of photos: https://www.kaggle.com/datasets/emmarex/plantdisease.
